# Chest Wall Reconstruction in Male Poland Syndrome

**Published:** 2010-12-13

**Authors:** Aditya Sood, Naveen Ahuja

**Affiliations:** ^a^Chicago Medical School, North Chicago, Illinois; ^b^Division of Plastic Surgery, Department of Surgery, New Jersey Medical School-University of Medicine and Dentistry of New Jersey, Newark

**Figure:**
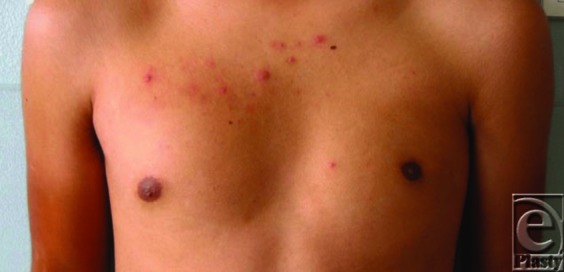


## DESCRIPTION

A 16-year-old boy with an unremarkablemedical history presented with complaints of chest asymmetry present since birth. Patient has no other complaints, including pain, decreased strength, or restricted range of motion.

## QUESTIONS

1. What is Poland syndrome?
2. How does this condition present and how is it diagnosed?
3. What are the treatment options?

## DISCUSSION

Poland syndrome is a sporadic, congenital condition with a wide spectrum of presentation. Absence of the sternocostal head of the pectoralis major muscle is considered pathognomonic for the condition. There is also an association with ipsilateral chest wall and limb derangements, the most common being syndactyly. The severity of Poland syndrome differs from person to person and is often not diagnosed or reported. The causes are unknown, although an interruption of the embryonic blood supply to the subclavian arteries around the 46th day of development is the prevailing theory.

Diagnosis of Poland syndrome is clinical, as the deficiencies presented are largely cosmetic. The most common (simple) form presents with a unilateral absence of the sternocostal head of the pectoralis majorwith subclavicular hollowing and a deficient axillary fold. Other common findings include axillary webbing that can be seen on arm abduction and elevation, smaller breast/chest with the nipple-areola complex displaced toward the axilla, shortening of the hemithorax, upper extremity shortening, aberrant chest hair distribution in males on the affected side, and syndactyly. Systemic findings may be present, especially in the more severe form of Poland syndrome. The simple form may be known to the patient but remain unannounced to family members until early adolescence when the patient becomes more conscious of the asymmetry. The severe form, however, is usually noted during infancy by the parents because the chest wall is quite visibly asymmetrical and a brachysyndactyly may also be present.

The surgical options for chest wall asymmetry depend on anatomical severity, gender, associated anomalies, and patient preferences. The understanding of the intervention options are complicated by the lack of detailed, long-term studies. Treatment options include autologous fat injection, pedicled latissimus dorsi muscle transfer, transverse rectus abdominus musculocutaneous flaps, deep inferior epigastric artery perforator flaps, custom-made chest wall prosthesis, nipple-areola complex repositioning, mammary prosthesis, sternal/rib reconstruction, contralateral operations (to ameliorate asymmetry), or a combination of these techniques.

**Figure 1:**
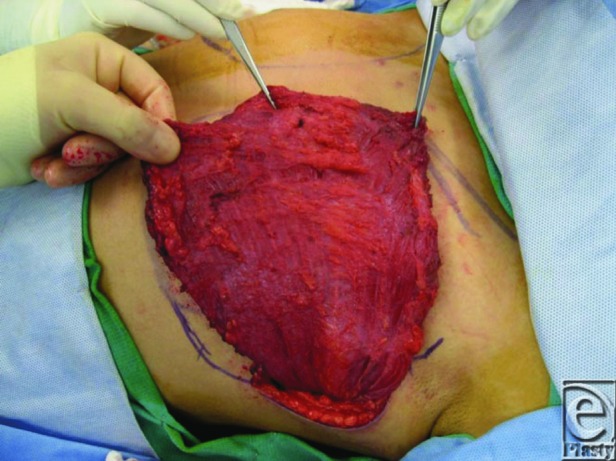
The left latissimus dorsi was mobilized and tunneled through an incision along the anterior axillary line.

**Figure 2:**
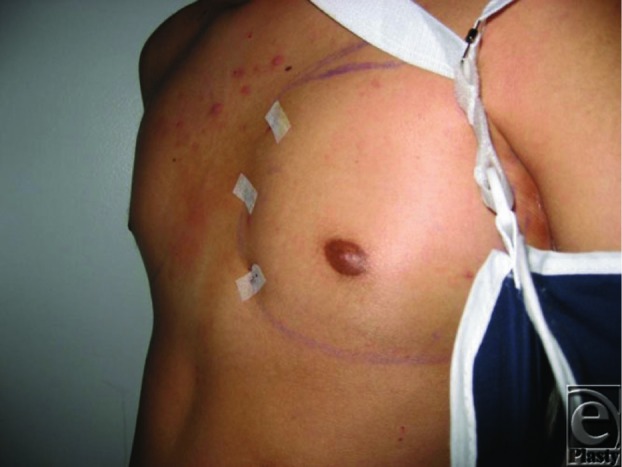
Postoperative day two. Note restoration of chest contour.

In the case described earlier, left chest fat transposition was performed with approximately 100 cc of autologous fat infiltrated into the left chest with reasonable contour at the completion of the procedure. Three months later, however, much of the fat graft resorbed and the asymmetry again became pronounced. A left latissimus dorsi pedicled flap was then performed. The ipsilateral latissimus muscle was mobilized and tunneled through an incision along the anterior axillary line (Fig 1). Three sutures were placed as secure anchorage points for the muscle to the left anterior chest wall along the sternal border. The postoperative course was uncomplicated, and the patient was satisfied with the increased definition of his left chest wall (Fig 2).

Complications of this procedure may include bleeding, infection, seroma formation, and wound dehiscence. This procedure leads to a lack of volume of the posterior axillary fold and ipsilateral back due to the absence of the latissimus dorsi. There is also a risk of postoperativeweakening of the shoulder girdle. The simple deformity of Polands syndrome in males is effectively effaced with a latissimus dorsi transfer, in accordance with Seyfur et al. It is important to note that the complex deformity may require furthermusculoskeletal repair.

## References

[1] Pereira LH, Sabatovich O, Santana KP, Picanco R, Sternodimas A (2008). Surgical correction of Poland's syndrome in males—a purposely designed implant. J Plast Reconstr Aesthet Surg.

[2] Pinsolle V, Chichery A, Grolleau JL, Chavoin JP (2008). Autologous fat injection in Poland's syndrome. J Plast Reconstr Aesthet Surg.

[3] Seyfer AE, Icochea R, Graeber GM (1988). Poland's anomaly: natural history and long-term results of chest wall reconstruction in 33 patients. Ann Surg.

[4] Seyfer AE, Fox JP, Hamilton CG (2010). Poland syndrome: evaluation and treatment of the chest wall in 63 patients. Plast Reconstr Surg.

